# Perfect Absorption and Reflection Modulation Based on Asymmetric Slot-Assisted Gratings without Mirrors

**DOI:** 10.3390/nano13222922

**Published:** 2023-11-09

**Authors:** Sangjun Lee, Sangin Kim

**Affiliations:** 1 Department of Electrical and Computer Engineering, Ajou University, Suwon 16499, Republic of Korea; lsjkmjh@ajou.ac.kr; 2 Department of Intelligence Semiconductor Engineering, Ajou University, Suwon 16499, Republic of Korea

**Keywords:** perfect absorption, mirror-less, graphene, tunable absorption, modulation

## Abstract

As a perfect graphene absorber without any external mirrors, we proposed asymmetric slot-assisted grating structures supporting two degenerate resonant modes of the guided-mode resonances (GMR) and the quasi-bound states in the continuum (quasi-BIC). The GMR mode functions as an internal mirror in conjunction with the background scattering, while the quasi-BIC, which is responsible for perfect graphene absorption, stems from the horizontal symmetry breaking by an asymmetric slot. By properly shifting the slot center from the grating center, the leakage rate of quasi-BIC can be controlled in such a way as to satisfy the critical coupling condition. We provide a comprehensive study on the coupling mechanism of two degenerate resonant modes for a one-port system mimicking the resonance. We also numerically demonstrated that our proposed grating structures show an excellent reflection-type modulation performance at optical wavelength ranges when doped double-layer graphene is applied. Due to the perfect absorption at the *OFF* state, a high modulation depth of ~50 dB can be achieved via a small Fermi level variation of ~0.05 eV. To obtain the lower insertion loss at the *ON* state, the higher Fermi level is required to decrease the graphene absorption coefficient.

## 1. Introduction

Over the past decade, there has been significant research focused on graphene, positioning it as a promising candidate for high-speed photodetectors [1,2,3,4,5] and modulators [6,7,8,9] due to its exceptional carrier mobility. Undoped mono-layer graphene, despite its thickness of ~0.34 nm, exhibits a consistent absorption efficiency of ~2.3% across various wavelengths, from visible to THz. However, there is a significant demand for enhanced graphene absorption to fulfill the needs of practical high-performance photodetectors. Our primary focus is on achieving the perfect absorption of an incident wave under single-sided illumination. Most perfect absorbers fall into one of the following three categories: the single-mode/mirror scheme [6,7,8,10,11,12,13,14,15,16,17], the dual-mode (or degenerate critical coupling) scheme [18,19,20], and the triple-mode scheme [21]. The most straightforward approach to achieving perfect absorption is the single-mode/mirror scheme, which involves a one-port resonant system with an external mirror on the backside to ensure zero transmission [6,7,8,10,11,12,13,14,15,16]. To achieve perfect absorption in this scheme, we must satisfy the ‘critical coupling condition (*γ_leak_* = *γ_loss_*)’, which means balancing the external leakage rate (*γ_leak_*) with the internal loss rate (*γ_loss_*) of the resonator. For the external 100% mirror, we can use either a distributed Bragg reflector (DBR) [10,11] or a metal mirror [6,7,8,12,13,14,15,16]. However, DBR, composed of a multilayer dielectric stack, requires a sophisticated growth technique that is limited to specific material systems [5], and a metal mirror inevitably introduces ohmic loss, which is undesirable for applications like photodetectors. The dual-mode scheme, while avoiding the use of a mirror, poses significant challenges in simultaneously meeting the frequency degeneracy and the critical coupling conditions of dual modes [18,19,20]. On the other hand, the triple-mode scheme simplifies the design compared to the dual-mode scheme but requires a somewhat complex fabrication process due to the addition of a slab structure [21].

Recently, our research group has introduced a novel approach inspired by the concept of a one-port system mimicking, leading to the development of some mirrorless perfect absorbers. These absorbers are based on an asymmetric single resonator supporting two degenerate resonant modes, in which only one mode experiences loss while the other mode acts as an internal reflector in conjunction with background scattering [22,23,24,25]. The asymmetric resonator can be easily implemented with a vertically asymmetric slab-waveguide grating (SWG) [22,23,24,25]. In this approach, a guided-mode resonance (GMR), which is of a relatively low-quality factor, was employed to function as the internal mirror, and the other resonant mode, which is responsible for absorption and of a relatively high-quality factor, could be another GMR mode of a higher diffraction order [22,23] or a quasi-bound states in the continuum (quasi-BIC) [24,25]. When a high-order diffraction GMR mode is employed, it is required that the index of the substrate should be lower than half of that of the grating (i.e., *n_SWG_* > 2*n_Sub_*). Whereas, in the quasi-BIC-based structures, the index requirement is not necessary, but there is another limitation; that is, the wave should be launched obliquely since the normal incidence of the wave cannot excite the BIC mode in the SWG.

**Figure 1:**
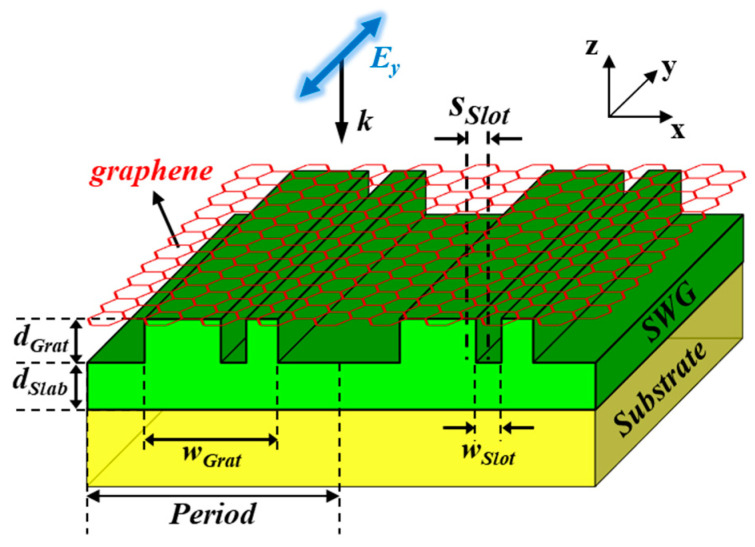
Schematic of the proposed perfect absorber, where undoped mono-layer graphene of 0.34 nm thickness as an absorbing medium is located on a slot-assisted SWG (slab-waveguide grating) consisting of slab and grating. Key parameter *s_Slot_* is defined as the distance between centers of SWG and shifted slot. The refractive indices of SWG and substrate are *n_SWG_* = 2.00 (Si_3_N_4_) and *n_Sub_* = 1.45 (SiO_2_), respectively.

In this work, we propose a novel quasi-BIC-based perfect absorber allowing a normal incidence angle, which incorporates an asymmetric slot into a SWG as depicted in Figure 1. To manipulate the quasi-BIC, our proposed absorber introduces structural asymmetry in the horizontal direction through an asymmetric slot, eliminating the need for an additional experimental setup to control the incidence angle, as opposed to the structure described in ref. [24]. By adjusting the position of the slot, we can control the leakage rate of quasi-BIC to satisfy the critical coupling condition. The proposed structure is still based on the one-port system mimicking concept, and we provide a comprehensive study on the coupling mechanism involving two degenerate resonant modes; that is, a quasi-BIC and a GMR mode.

Previously, similar grating structures with symmetry-breaking slots for quasi-BIC-based normal incidence perfect absorption have been reported [26,27,28]. Sang et al. proposed a perfect graphene absorber using quasi-BIC in an asymmetric slot-assisted photonic crystal slab (PCS), in which the slot is located within the slab region rather than the ridge, and mono-layer graphene is sandwiched between the slab and substrate [26]. In ref. [27,28], two asymmetric dielectric gratings are employed to support the quasi-BIC with mono-layer graphene (or borophene) placed between these two gratings. In those works, it is not clear how to achieve zero transmission, which is indispensable to achieve perfect absorption for single-sided illumination in two-port systems. The quasi-BIC, which is responsible for absorption (or loss), cannot achieve zero transmission by itself, requiring another resonant mode in the structure or an external reflector. The clear analysis on this, and thus, their systematic design processes, have not been provided. Additionally, those structures require a more intricate fabrication process since the absorbing layer (graphene or borophene) is located beneath or in the middle of the grating structure. Therefore, in this work, we revisit the quasi-BIC-based normal incidence perfect absorber design problem, offering systematic design process of the slot-assisted grating perfect absorber that requires a relatively easier fabrication process.

Moreover, in our proposed structure, when we replace the top single layer of undoped graphene with doped double-layers of graphene, an outstanding reflection-type optical modulator can be implemented within the optical wavelength range (*λ* = 1540–1570 nm). Perfect absorption is achieved in the *OFF* state (*R* < 0.001%), and a substantial modulation depth of ~50 dB is attainable with a minimal Fermi level variation of ~0.05 eV. To minimize insertion loss in the *ON* state, a higher Fermi level is necessary to reduce the absorption coefficient of graphene. To examine the reflection and absorption spectra, as well as the field profiles of various resonant modes in our proposed absorber and modulator, we employ a rigorous coupled-wave analysis (RCWA) simulation [29] using commercial software called DiffractMOD (Version 8.2.0.3, Synopsys Inc., Sunnyvale, CA, USA).

## 2. Perfect Absorption in Asymmetric Slot-Assisted SWG

### 2.1. Structure of the Proposed Perfect Absorber

The proposed absorber (Figure 1) consists of a one-dimensional (1-D) SWG of Si_3_N_4_ stacked on SiO_2_ substrate, where the grating (ridge) and the slab (sublayer) share the same index, and an undoped (*E_f_* = 0 eV) mono-layer graphene is placed just above the SWG. The refractive indices for Si_3_N_4_ and SiO_2_ are 2.00 and 1.45, respectively (i.e., *n_SWG_* = *n_Slab_* = *n_Grat_* = 2.00, *n_Sub_* = 1.45). These dielectric materials are assumed to have no loss. The asymmetric slot incorporated into the SWG has the same thickness as the ridge. *s_Slot_* represents the distance between the centers of the SWG and the shifted slot. It is worth noting that while the inclusion of asymmetric slots in grating structures has also been discussed in refs. [30,31,32,33,34], these reports did not employ the one-port system mimicking concept. To determine the permittivity of graphene, Kubo formulation [21,35] was utilized, with parameters including a graphene thickness of 0.34 nm, a Fermi velocity of 10^6^ m/s, and a mobility of 0.5 m^2^/Vs. The light of transverse electric (TE) polarization, whose electric field is perpendicular to the incident plane, is incident normally from the air.

### 2.2. Coupling of Two Degenerate Resonant Modes

**Figure 2:**
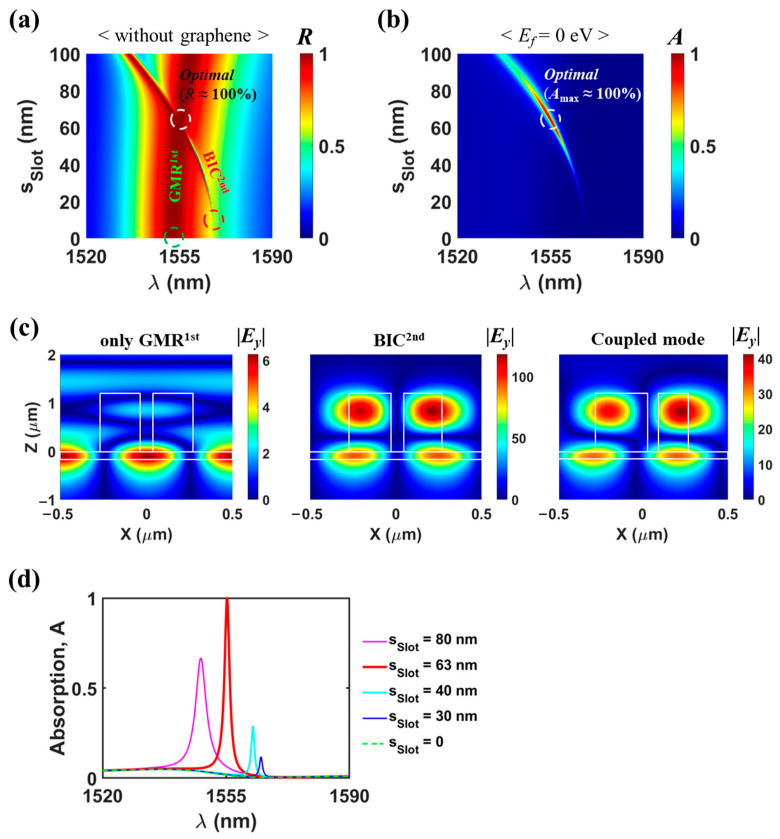
(**a**) Reflection map as a function of *s_Slot_* when only the graphene is removed in the optimized absorber. (**b**) Absorption map as a function of *s_Slot_* in the optimized graphene perfect absorber with *E_f_* = 0 eV. (**c**) (**Left**), (**middle**), and (**right**) panels indicate the normalized electric field distribution (|*E_y_*|) at three points marked by the open green circle (*s_Slot_* = 0 and *λ* = 1552.5 nm), open red circle (*s_Slot_* = 10 nm and *λ* = 1567.62 nm), and open white circle (the optimal condition of *s_Slot_* = 63 nm and *λ* = 1555.3 nm) in (**a**), respectively. The white boxes indicate the interface between different materials in the slot-assisted SWG. (**d**) Absorption spectra for *s_Slot_* = 0, 30, 40, 63, and 80 nm, which are extracted from (**b**). In all the calculations, *Period* = 1000 nm, *d_Slab_* = 190 nm, *w_Grat_* = 550 nm, *d_Grat_* = 1183 nm, *w_Slot_* = 63 nm.

We optimized the proposed absorber to achieve perfect absorption (*A* > 99.9%) at near *λ* = 1.55 μm for undoped mono-layer graphene. The optimal design parameters are as follows: *Period* = 1000 nm, *d_Slab_* = 190 nm, *w_Grat_* = 550 nm, *d_Grat_* = 1183 nm, *w_Slot_* = 63 nm, and *s_Slot_* = 63 nm. Figure 2a,b show reflection (*R*) and absorption (*A*) maps as a function of *s_Slot_* for the optimal structure without graphene and with graphene, respectively. A sharp reflection branch corresponds to BIC^2nd^, while a broadband (or flat-top) reflection branch relates to GMR^1st^. The modal order (such as first and second) indicates the number of the peak points in the field profile in the vertical direction. The main absorption peak locus in Figure 2b aligns with the sharp reflection peak branch of BIC^2nd^ in Figure 2a. This observation suggests that BIC^2nd^ primarily contributes to graphene absorption. More importantly, a perfect absorption is achieved at the intersecting point of the BIC^2nd^ and the GMR^1st^ branches (in detail, optimal condition of *s_Slot_* = 63 nm and *λ* = 1555.3 nm).

For the structures with horizontal symmetry, the normal incident wave can be phase-matched to two leaky modes counter-propagating along the waveguide grating structure. Strong coupling of two leaky guided modes induces constructive and destructive interference. The former produces a leaky mode (called GMR [36,37,38,39]) with strong radiation losses, and the latter leads to an ideal BIC without radiative losses [38,39]. The GMR and BIC have symmetric (horizontally even) and anti-symmetric (horizontally odd) field configurations, respectively. The ideal BIC is well-known as a special eigenstate that remains localized and possesses an infinite quality factor (*Q*-factor) even though it exists within the light cone of the surrounding medium [40,41]. In practice, the BIC can be realized as a quasi-BIC whose finite *Q*-factor is adjusted by breaking structural symmetries [19,42,43,44,45,46,47] or through parameter tuning [48,49,50,51,52]. In the context of our proposed absorber, the BIC is symmetry-protected [19,24,42,43,44,45,46,47]. Indeed, when *s_Slot_* = 0 (i.e., the absence of horizontal asymmetry), only a GMR^1st^ is excited since the excitation of the ‘symmetry-protected BIC mode’ is explicitly prohibited. Regardless of *s_Slot_*, GMR^1st^ exhibits a relatively consistent and lower quality factor (*Q* ~ 50) and brings about a broadband or flat-top reflection spectrum in conjunction with the Fabry–Perot-like background scattering [22,23,24]. Conversely, the reflection bandwidth of BIC^2nd^ increases with an increase in *s_Slot_*, as observed in Figure 2a. Therefore, by adjusting the slot center position, the leakage rate of quasi-BIC can be controlled such that the critical coupling condition is satisfied. The left, middle, and right panels in Figure 2c display the normalized electric field distribution (|*E_y_*|) at three points marked by the open green circle (only GMR^1st^ at *s_Slot_* = 0, *λ* = 1552.5 nm), the open red circle (BIC^2nd^ at *s_Slot_* = 10 nm, *λ* = 1567.62 nm), and the open white circle (the coupled mode caused by the coupling of GMR^1st^ and BIC^2nd^ at the optimal point) in Figure 2a, respectively. The relevant mode at *s_Slot_* = 10 nm has a symmetric field configuration, and it can be regarded as only BIC^2nd^ because the effect of GMR^1st^ is negligibly weak. The enhanced electric fields of the coupled mode at the optimal point strongly resemble those of BIC^2nd^, covering both the ridge and slab regions, providing clear evidence that BIC^2nd^ is dominantly responsible for graphene absorption, whereas the electric fields of GMR^1st^ (which has a symmetric configuration) are concentrated in slab (not ridge) region.

Furthermore, the absorption spectra at *s_Slot_* = 0, 30, 40, 63, and 80 nm are also illustrated in Figure 2d, which are extracted from Figure 2b. For *s_Slot_* = 63 nm (as the optimal slot position), one-port resonant system mimicking (i.e., virtual one-port resonant system [22,23,24]) is induced through the proper indirect coupling between GMR^1st^ and BIC^2nd^, facilitated by the appropriately designed partial reflection within the internal wave propagation channel. This setup enables the achievement of perfect absorption via critical coupling, even without the use of external mirrors. However, in the remaining cases, the absorber structures can be treated as the single-mode resonator without external mirror because GMR^1st^ and BIC^2nd^ are either separated or weakly coupled. Consequently, achieving perfect absorption is impossible under these conditions.

### 2.3. Validation of the One-Port Resonant System Mimicking

**Figure 3:**
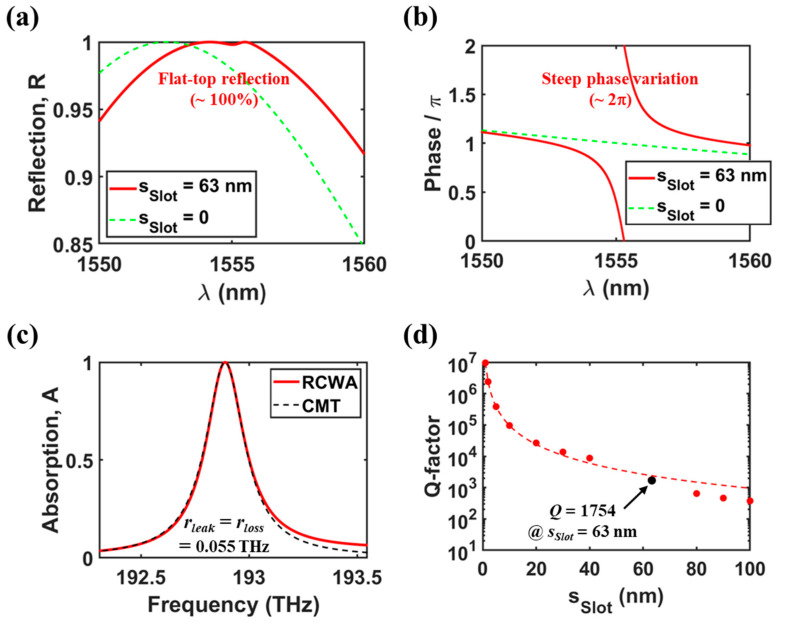
(**a**) Reflection and (**b**) its phase spectra for *s_Slot_* = 0 and 63 nm when only the graphene is removed in the optimized absorber. (**c**) Absorption spectra obtained by RCWA calculation and CMT fitting in the optimized absorber with *E_f_* = 0 eV. (**d**) *Q*-factors of the coupled mode (red solid circles) as a function of *s_Slot_* when only the graphene is removed in the optimized absorber, which are derived by fitting Fano formula to the reflection spectra in Figure 2a. The single black solid circle indicates the *Q*-factor at *s_Slot_* = 63 nm, which is derived from (**c**). The red dashed curve is for a proper inverse quadratic equation (*Q* = 31.6 × (*s_Slot_*/*w_Grat_*)^−2^) as approximate expression. All the remaining parameters are same as those of Figure 2.

To validate the one-port resonant system mimicking in our optimized absorber structure without graphene, we first examine the reflection and its phase spectra for *s_Slot_* = 0 and 63 nm at *λ* = 1550–1560 nm, as shown in Figure 3a,b, respectively. For *s_Slot_* = 0, the observation of flat-top reflection and gradual phase variation can be attributed only to the presence of low-Q GMR^1st^. In contrast, for *s_Slot_* = 63 nm, near the resonance wavelength, the flat-top reflection close to 100% is maintained, and simultaneously, the phase varies steeply by ~2π. The reflection characteristics resemble those of a phase shifter based on an ideal asymmetric Fabry–Perot cavity with a perfectly reflecting bottom mirror [53,54,55]. Therefore, it can be stated that the designed structure mimics the system composed of a single-mode resonator and an external mirror by behaving like a virtual one-port resonant system. According to the coupled mode theory (CMT), absorption efficiency in a lossy one-port resonant system can be described by the following equation [10,24,25]:

(1) $$A(\omega )=\frac{4\gamma_{leak}\gamma_{loss}}{{\left(\omega -\omega_{o}\right)}^{2}+{\left(\gamma_{leak}+\gamma_{loss}\right)}^{2}}$$

where *ω_o_*, *γ_leak_*, and *γ_loss_* are the resonant frequency, leakage rate, and loss rate, respectively. Figure 3c shows the absorption spectra by the RCWA calculation and the CMT fitting of Equation (1) for the optimized absorber structure with graphene. The excellent agreement between the RCWA and the CMT results suggests that the considered structure behaves like a lossy one-port resonant system. The fitting parameters are as follows: *γ_leak_* = *γ_loss_* = 0.055 THz, *w_o_* = 192.89 (*λ_o_* = 1555.3 nm), and finally *Q* = *w_o_*/2/(*γ_leak_* + *γ_loss_*) = 877.

**Figure 4:**
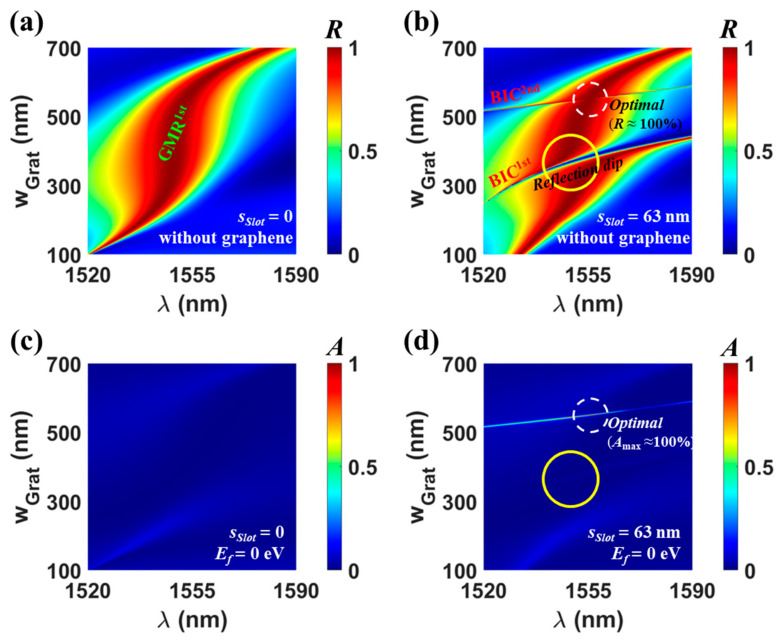
Reflection map as a function of *w_Grat_* for (**a**) *s_Slot_* = 0 and (**b**) *s_Slot_* = 63 nm when only the graphene is removed in the optimized absorber. Absorption map as a function of *w_Grat_* for (**c**) *s_Slot_* = 0 and (**d**) *s_Slot_* = 63 nm in the optimized graphene perfect absorber (*E_f_* = 0 eV). All the remaining parameters are same as those of Figure 2.

We also investigated the quality factor of the relevant resonant mode as a function of *s_Slot_* for our optimized structure without graphene, as shown in Figure 3d. The red solid circles represent the coupled (or hybrid) mode based on the indirect coupling between GMR^1st^ and BIC^2nd^, and they are obtained by fitting Fano formula to the reflection spectra of Figure 2a. The transmission in the Fano formula can be described as follows [56,57,58,59,60]:

(2) $$T(\omega )=\frac{T_{o}}{{1+q}^{2}}\frac{{\left[q+(\omega -\omega_{o})/\gamma_{leak}\right]}^{2}}{{1+[(\omega -\omega_{o})/\gamma_{leak}]}^{2}}\;\;+T_{bg}(\omega )$$

where *q* is the Fano asymmetry parameter, *T_bg_* and *T_o_* are the background contribution of non-resonant modes to the resonant peak amplitude and the offset, respectively. For example, for *s_Slot_* = 30 nm, the fitting parameters are as follows: *γ_leak_* = 0.007 THz, *w_o_* = 191.7043 (*λ_o_* = 1564.91 nm), *q* = −1.32, *T_o_* = 0.81, and finally *Q* = *w_o_*/2/*γ_leak_* = 13,693. In the range of ~40 nm < *s_Slot_* < ~80 nm, which is around the optimal point, the fitting data were not included due to the inadequate match of Fano formula, caused by the strong coupling effect between two resonant modes. In general, Fano resonances at two-port resonant system are characterized by asymmetric or symmetric spectral line profiles owing to the destructive interference at the reflection or transmission port [56,57,58,59,60,61,62,63]. However, they never represent the flat-top reflection profile with steep phase variation (that is, virtual one-port resonant system), which stems from the indirect coupling effect between two resonant modes. The red dashed curve represents the estimated *Q*-factor of only (or separated) BIC^2nd^, which can be obtained by a proper inverse quadratic equation of *Q* = 31.6 × (*s_Slot_*/*w_Grat_*)^−2^ as approximation expression [56,58,59]. For small value of *s_Slot_* (<~20 nm), the coupled mode is actually the same as only BIC^2nd^, and thus the quadratic dependence of the *Q*-factor (or bandwidth) on *s_Slot_* is confirmed. For a larger *s_Slot_*, the *Q*-factor begins to deviate from the curve of only BIC^2nd^. This phenomenon appears to be caused by the coupling effect between GMR^1st^ and BIC^2nd^, as well as the significant distortion of BIC^2nd^ by larger structural asymmetry. As seen in the single black solid circle on Figure 4d, we also included the *Q*-factor of the coupled mode at the optimal value of *s_Slot_* = 63 nm (in detail, *Q* = *w_o_*/2/*γ_leak_* = 192.89/2/0.055 = 1754, which is derived from Figure 3c).

### 2.4. Two Types of Indirect Coupling Conditions

For an additional analysis on the indirect coupling of two degenerate resonant modes, as shown in Figure 4, the *w_Grat_* dependency of reflection (top panels) and absorption (bottom panels) at *s_Slot_* = 0 (left panels) and *s_Slot_* = 63 nm (right panels) is investigated for optimal structures without and with undoped graphene. For *s_Slot_* = 0 nm, absorption is inevitably poor because single mode (i.e., low-Q GMR^1st^) exists without external mirrors, as seen in Figure 4a,c. For *s_Slot_* = 63 nm, two additional branches of BIC^1st^ and BIC^2nd^ appear on the reflection map (see Figure 4b). The relevant modes correspond to quasi-BICs due to asymmetric slots, which are manifested by breaking the symmetry of a symmetry-protected BIC with an infinite *Q*-factor. The appearance of different quasi-BICs depending on *w_Grat_* originates from phase-matching to different leaky modes along the SWG. When *w_Grat_* is very large or small, the main reflection branch of GMR^1st^ becomes very narrow due to the weak scattering strength of the ridge part. For moderate *w_Grat_* compared to *Period*, the BIC^1st^ and BIC^2nd^ of high-*Q* reflection branches cross different regions of the broadband reflection branch due to GMR^1st^ as follows: in detail, the intersecting regions marked by the yellow open circle and the white dashed circle, respectively. In particular, the optimal point in the white dashed circle (*s_Slot_* = 63 nm, *w_Grat_* = 550 nm, and *λ* = 1555.3 nm) is the same as that in Figure 2a. As already demonstrated in Figure 2, the introduction of sharp BIC^2nd^ does not distort the flat-top reflection property of GMR^1st^ and thus mimics a one-port resonant system. As a result, when the mono-layer graphene is introduced, the absorption at the crossing region relevant to BIC^2nd^ is remarkably strong (see the white dashed circle in Figure 4d). However, the reflection branch of BIC^1st^ shows a sharp Fano line shape (i.e., reflection dip) at the crossing region, which does not follow the one-port resonant system mimicking concept, like the usual case of refs. [33,61,62,63]. So, absorption is very low (see the yellow open circle in Figure 4d), and it is impossible to achieve perfect absorption even if the graphene position is changed. This result suggests that the perfect reflection or perfect transmission at the resonance depends on the indirect coupling condition of two degenerate modes. Since the indirect coupling stems from the partial reflections at the surfaces of the SWG, the coupling condition is strongly dependent on *w_Grat_* [22,23,24]. The specific indirect coupling condition can be derived by the CMT in refs. [22,23,24] because the proposed SWG absorber can be also modeled as an asymmetric single resonator coupled to two external ports. Moreover, it is notable that BIC^2nd^ corresponds to a vertically even mode, whereas BIC^1st^ and GMR^1st^ have field configurations of vertically odd modes across the SWG. We surmise that the similarity in electric fields between quasi-BIC and GMR^1st^ is closely bound up with two types of indirect coupling conditions, which seems to be a rich topic of our future research.

### 2.5. Fabrication Error Tolerance

**Figure 5:**
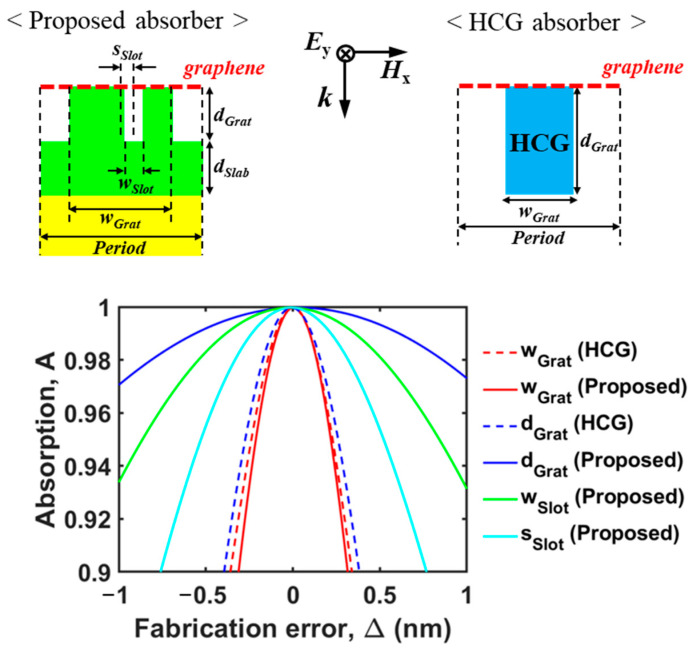
Fabrication tolerance in the several optimal parameters for the proposed perfect absorber (*Period* = 1000 nm, *d_Slab_* = 190 nm, *w_Grat_* = 550 nm, *d_Grat_* = 1183 nm, *w_Slot_* = 63 nm, *s_Slot_* = 63 nm, and *λ* = 1555.3 nm) and the HCG perfect absorber based on two degenerate resonant modes of opposite-symmetry (*Period* = 1000 nm, *w_Grat_* = 953 nm, *d_Grat_* = 291.4 nm, and *λ* = 1420.07 nm). The insets on the top show the 2D schematic of both perfect absorbers.

In this subsection, we discuss the fabrication tolerance of the proposed perfect absorber. As shown in Figure 5, we numerically investigated the absorption as a function of the fabrication error in several optimal parameters for the proposed perfect absorber (*Period* = 1000 nm, *d_Slab_* = 190 nm, *w_Grat_* = 550 nm, *d_Grat_* = 1183 nm, *w_Slot_* = 63 nm, *s_Slot_* = 63 nm, and *λ* = 1555.3 nm) and the HCG (high-contrast grating) perfect absorber based on two degenerate resonant modes of opposite-symmetry [18] (*Period* = 1000 nm, *w_Grat_* = 953 nm, *d_Grat_* = 291.4 nm, and *λ* = 1420.07 nm when *n_HCG_* = 3.40). Compared to the HCG scheme, our proposed scheme demonstrates a ~5 times larger fabrication error tolerance in terms of *d_Grat_*, while showing a similar tolerance in terms of *w_Grat_*. Also, the fabrication error tolerance in terms of slot parameters (*w_Slot_* and *s_Slot_*) is larger than that of grating parameters (*w_Grat_* and *d_Grat_*) in the HCG scheme.

## 3. Reflection Modulation in Asymmetric Slot-Assisted SWG

### 3.1. Structure of the Proposed Reflection-Type Modulator

**Figure 6:**
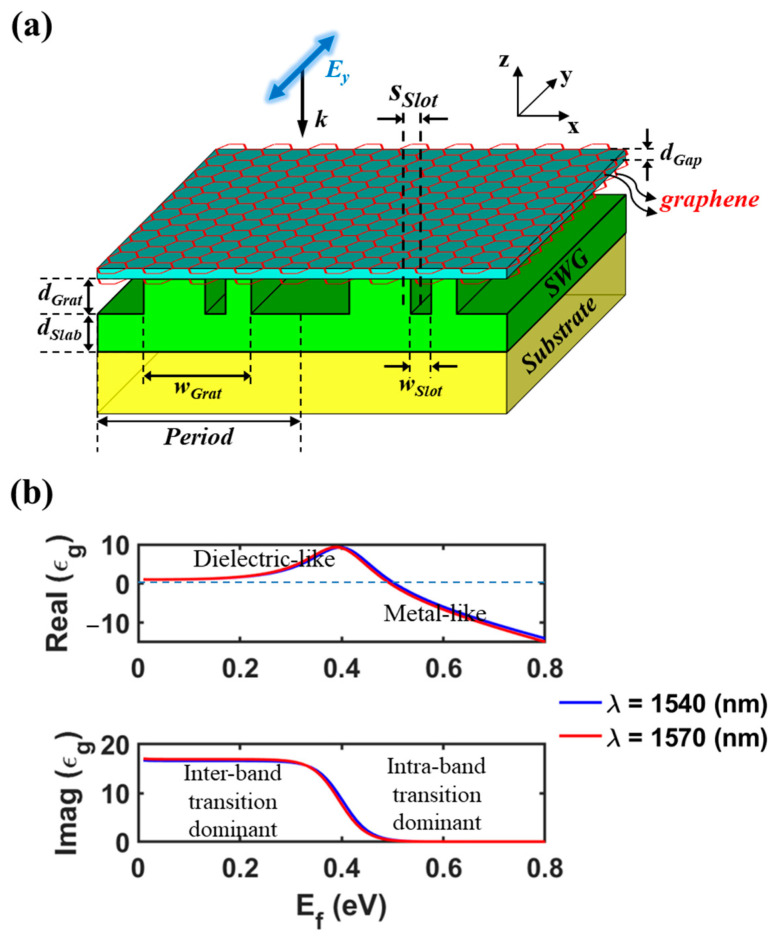
(**a**) Schematic of the proposed reflection modulator, where the gap layer enclosed by doped double-layer graphene is located on a slot-assisted SWG. The refractive indices of SWG, substrate, and gap are *n_SWG_* = 2.00 (Si_3_N_4_), *n_Sub_* = 1.45 (SiO_2_), and *n_Gap_* = 1.73 (Al_2_O_3_), respectively. (**b**) Real and imaginary part of permittivity (*ε_g_*) of graphene for two wavelengths over the Fermi level range of *E_f_* < 0.8 eV.

In this section, by leveraging the dynamically tunable absorption properties of doped double-layer graphene, we explore the proposed absorber structure as a novel reflection-type modulator without external mirrors. As a proof of concept, the reflection modulator structure of Figure 6a is proposed. The gap (acting as gate oxide) between the two monolayer graphene sheets is filled with a thin layer of Al_2_O_3_ (*n_Gap_* = 1.73, *d_Gap_* = 5 nm), which enables the electrostatic gate control. The chemical potential (that is, Fermi level *E_f_*) of graphene is modulated by a gate voltage across the gap. The remaining components of the reflection modulator are the same as the perfect absorber structure of Figure 1. Figure 6b shows the real and imaginary parts of the permittivity (*ε_g_*) of graphene at different wavelengths (*λ* = 1540 and 1570 nm) across the Fermi level range of *E_f_* < 0.8 eV, which are extracted from the Kubo formulation [21,35], with parameters including a graphene thickness of 0.34 nm, a Fermi velocity of 10^6^ m/s, and a mobility of 0.5 m^2^/Vs. Notably, within the range of 0.20 eV < *E_f_* < 0.60 eV, the loss rate (or absorption coefficient) of graphene rapidly decreases with *E_f_*, which is attributed to the shift in dominant contribution from inter-band transition to intra-band transition. For instance, at *E_f_* = 0.50 eV and 0.60 eV, the loss rate diminishes to ~1/45 and ~1/330 of the value observed for relatively lowly doped graphene (*E_f_* < 0.2 eV), respectively.

### 3.2. Excellent Reflective Modulation Performance

**Figure 7:**
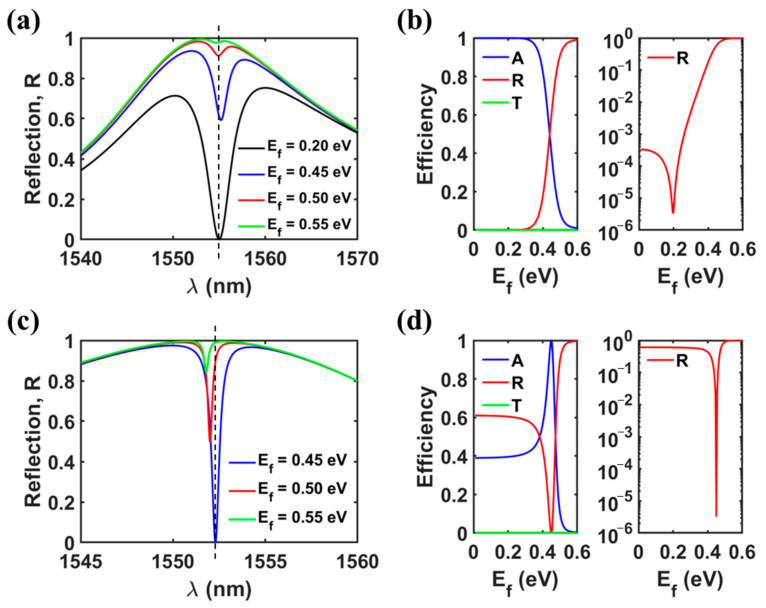
(**a**) Reflection spectra at different Fermi levels, and (**b**) reflection at the optimal wavelength of *λ* = 1555 nm as a function of Fermi level, when a reflection modulator is designed in such a way as to achieve the perfect absorption condition (as the *OFF* state) at *E_f_* = 0.20 eV (*Period* = 1000 nm, *d_Slab_* = 190 nm, *w_Grat_* = 550 nm, *d_Grat_* = 1237 nm, *w_Slot_* = 71 nm, and *s_Slot_* = 77 nm). (**c**) Reflection spectra at different Fermi levels, and (**d**) reflection at the optimal wavelength of *λ* = 1552.3 nm as a function of Fermi level, when a reflection modulator is designed in such a way as to achieve the perfect absorption condition (as the *OFF* state) at *E_f_* = 0.45 eV (*Period* = 1000 nm, *d_Slab_* = 190 nm, *w_Grat_* = 550 nm, *d_Grat_* = 1109 nm, *w_Slot_* = 69 nm, and *s_Slot_* = 34.4 nm).

We first conducted optimization for the reflection modulator to achieve perfect absorption at *E_f_* = 0.20 eV, designating this condition as the *OFF* state. The optimized design parameters are as follows: *Period* = 1000 nm, *d_Slab_* = 190 nm, *w_Grat_* = 550 nm, *d_Grat_* = 1237 nm, *w_Slot_* = 71 nm, and *s_Slot_* = 77 nm). We define the *ON* state as the high reflection state and the *OFF* state as the low reflection state. In Figure 7a, a reflection dip near zero is depicted, resulting from the perfect absorption at *E_f,OFF_* = 0.20 eV and *λ* = 1555 nm. The reflection at the optimal wavelength of 1555 nm (indicated by the white dashed line) increases with rising *E_f_*, eventually approaching 100%. To achieve a lower insertion loss (*IL*), defined as −10·log(*R_ON_*), where *R_ON_* is the reflection at the *ON* state, a higher *E_f,ON_* is necessary due to the lower loss rate of graphene. For example, *IL* = 2.15 dB, 0.40 dB, and 0.09 dB at *E_f,ON_* = 0.45 eV, 0.50 eV, and 0.55 eV, respectively. In contrast to other reflection modulator discussed in refs. [6,7,8], our proposed modulator employs a one-port resonant system mimicking concept instead of utilizing a metal reflector. Conventional modulators using novel metal reflectors inevitably experience high ohmic losses, especially in the visible or near-IR range. However, the proposed modulator is expected to exhibit a significantly lower insertion loss due to the absence of metal. For a more intuitive grasp of the tunable property, Figure 7b presents the *E_f_* dependency of reflection at the optimal wavelength. The modulation depth (*MD*) is defined as 10·log(*R_ON_*/*R_OFF_*), where *R*_OFF_ is the reflection at the *OFF* state. *MD* > 50 dB if *E_f,ON_* = 0.50 eV as the *ON* state. The excellent modulation performance is attributed to the presence of perfect absorption (or extremely low *R_OFF_*).

An improved modulation performance can be achieved by optimizing the reflection modulator to meet the perfect absorption condition at higher *E_f,OFF_*. For example, we can select *E_f,OFF_* = 0.45 eV, for which the optimal design parameters are as follows: *Period* = 1000 nm, *d_Slab_* = 190 nm, *w_Grat_* = 550 nm, *d_Grat_* = 1109 nm, *w_Slot_* = 69 nm, and *s_Slot_* = 34.4 nm. As shown in Figure 7c,d, with *E_f,ON_* = 0.50 eV, we attain a modulation depth exceeding 50 dB via a much smaller *E_f_* variation (Δ*E_f_* = 0.50 eV − 0.45 eV = 0.05 eV) in comparison to former case (Δ*E_f_* = 0.50 eV − 0.20 eV = 0.30 eV). This improved modulation performance is attributed to an increase in *Q*-factor of dominant resonant mode (BIC^2nd^). The resonance properties of high-*Q* mode are more sensitive to material index. In details, for the latter modulator, *Q* = *λ*/Δ*λ* = 1552.3 nm/0.51 nm ≈ 3044, whereas for the former modulator, *Q* = 1555 nm/3.7 nm ≈ 420.

## 4. Summary and Discussion

In this work, we proposed novel perfect graphene absorbers and reflection-type modulators featuring two degenerate resonant modes; implementing the concept of a one-port system mimicking, we conducted numerical simulation to demonstrate their performance and the coupling mechanism of the relevant resonant modes. One of these modes is the quasi-BIC, which is activated due to the asymmetry of the slot within the SWG and plays a dominant role in achieving perfect absorption. The other mode is the GMR, which acts as an internal mirror, working in concert with background scattering. By adjusting the position of the slot relative to the grating center, it is possible to regulate the leakage rate of quasi-BIC to achieve satisfaction of the critical coupling condition. When doped double-layer graphene is applied, due to dynamically tunable absorption, an excellent reflection modulation performance can be obtained at optical wavelength ranges. For instance, when we set *E_f,OFF_* = 0.45 eV and *E_f,ON_* = 0.50 eV, we attain a high modulation depth exceeding 50 dB with only a small variation in Fermi level of 0.05 eV, and the insertion loss is below 1 dB. Notably, our proposed structures eliminate the need for external mirrors, and the graphene layers can be transferred onto the SWG in the final fabrication step, simplifying the manufacturing process significantly. In addition, owing to the absence of ohmic loss in metals, our perfect absorber scheme is highly desirable for potential optical device applications such as photodetectors, modulators, switches, and sensors.

In our proposed devices, the suspended graphene layers are used, and the quality of graphene is an important factor for the performance of the proposed devices. It is well known that the quality of graphene grown by chemical vapor deposition (CVD) strongly depends on the substrate, and so far, graphene grown on the late transition metal substrate, such as Cu or Ni, shows the best quality, enabling large area monolayer graphene production as well [64]. So, the suspended graphene layers in the proposed devices can be formed by transferring graphene grown on the late transition metal. It has been experimentally demonstrated that the transfer process does not degrade the graphene quality in the fabrication of devices of similar structure to ours [65,66].

The fabrication of the proposed modulator requires two graphene transfer processes and the deposition of the thin layer of Al_2_O_3_ in between them. Recently, it has been shown that ultrathin uniform Al_2_O_3_ layers on graphene can be deposited using reversible hydrogen plasma functionalization followed by atomic layer deposition (ALD) [67]. So, the suspended graphene-Al_2_O_3_-graphene structure is fabricable.

When we control the chemical potential (*E_f_*) of graphene by applying a gate voltage (*V_G_*), they are related as *V_G_* = *qt*(*E_f_*)^2^/(*πħ*^2^*ε*(*V_f_*)^2^) from the simple capacitance model, where *t* and *ε* are the thickness and the permittivity of the Al_2_O_3_ layer, respectively, *ħ* is the reduced Plank constant and *V_f_* is the Fermi velocity [68]. So, assuming *V_f_* = 10^6^ m/s, which is a typical value in high-quality monolayer graphene, a chemical potential of *E_f_* = 0.5 eV requires a gate voltage of *V_G_* = ~1.35 V for *t* = 5 nm. This voltage corresponds to an electric field of ~2.7 MV/cm in the Al_2_O_3_ layer, which is below the breakdown field for Al_2_O_3_, ~5 MV/cm [69]. Although the Fermi velocity may not be the direct measure of the graphene quality, this calculation for the dynamic carrier density control assumes that the quality of monolayer graphene is high enough to guarantee the linear dispersion relation of electrons.

As mentioned in Section 2.5, the performance of the absorber can be negatively affected by inevitable fabrication errors. Additionally, the optical properties (loss) of graphene grown using the CVD may undergo changes due to unintentional doping during the growth or transfer process. This can lead to a disruption in the critical coupling condition, resulting in reduced absorption. To address this issue and recover from performance degradation caused by unforeseen variations in design parameters, there is a need for a way to adjust the properties of graphene. This adjustment can be achieved through the proposed modulator structure. In the demonstrated operation of this modulator, the voltage control allows intentional tuning of graphene properties, breaking the critical coupling condition when needed. Remarkably, this modulator structure can also serve as a highly efficient absorber with tolerance for fabrication errors.

## Data Availability

Data are contained within the article.
